# Fabrication and Performance Regulation of Lightweight Porous Electromagnetic Absorbing Materials via CO_2_ Nucleation-Free Foaming of EP

**DOI:** 10.3390/polym16243549

**Published:** 2024-12-19

**Authors:** Tienan Dong, Jingru Quan, Funing Huang, Yitong Guan, Zihong Lin, Zeyao Wang, Yuheng Liu, Zusheng Hang, Yupei Zhao, Yu’an Huang

**Affiliations:** 1 School of Petrochemical Engineering, Changzhou University, Changzhou 213164, China; dtn_chemistry@163.com; 2 School of Materials Science and Engineering, Nanjing Institute of Technology, Nanjing 211167, China; 220243290@seu.edu.cn (J.Q.); njithfn@163.com (F.H.); m18054502136@163.com (Y.G.); 15951855128@163.com (Z.L.); 13429541387@163.com (Z.W.); 15861609873@163.com (Y.L.); anhuzi@163.com (Y.H.)

**Keywords:** nucleation-free foaming, mesoporous wave absorption, loss coupling

## Abstract

In this study, CO_2_ reacted with a curing agent through nucleophilic addition to form ammonium salts, enabling the stable capture and internal release of CO_2_, which achieved gas-phase nucleation and foaming. Additionally, the introduction of wave-absorbing agents improved the absorption mechanism and promoted uniform foaming. This nucleation-free foaming process relies on the induced growth of gas nuclei and the synergistic effect of the wave-absorbing agents, effectively preventing the uneven foaming issues caused by traditional nucleating agents. Ultimately, a lightweight epoxy foam absorbing material (LFAM) was developed. BET tests showed that 2.0 wt% carbon-based wave-absorbing agents (LFAMs–A2) expanded the material’s volume to 4.6 times its original size, forming a uniform porous structure. VNA tests revealed that LFAMs–A2 achieved a minimum reflection loss of −13.25 dB and an absorption bandwidth of 3.7 GHz in the 12–18 GHz range. The material with 2.0 wt% ferrite-based wave-absorbing agents (LFAMs–C2) achieved a minimum reflection loss of −26.83 dB at 16.6 GHz and an absorption bandwidth of 5.3 GHz, nearly covering the Ku band. DSC tests indicated that the material maintained good thermal stability at 150 °C. This study provides a new approach for lightweight coatings and structural optimization, with broad application potential in 5G communications, microwave anechoic chambers, and aerospace fields.

## 1. Introduction

With the rapid development of modern electronic technology and informatization, electromagnetic pollution has become a global concern [1], driving an increasing demand for electromagnetic wave-absorbing materials. Traditional absorbing materials, such as metal oxides and ferrite-based composites, have been widely applied due to their excellent absorption performance in low-frequency bands [2,3,4,5]. However, these absorbers and composite matrices often suffer from high density, sizable thickness, and a narrow absorption bandwidth, making them unsuitable for lightweight and high-efficiency absorption scenarios [6,7]. This limitation hinders their broader application in fields such as aerospace, 5G communication, and wearable electronic devices [8].

To address these issues, researchers have increasingly focused on developing new lightweight wave-absorbing materials [9,10]. Ferrite wave-absorbing agents and carbon-based materials have gained significant attention due to their outstanding performance. Ferrite absorbers have achieved notable performance improvements through ion substitution and microstructure design [11,12,13]. For example, Mn–Zn ferrite plate absorbers fabricated using self-reactive jet-forming technology showed a minimum reflection loss (*RL_min_*) of −14 dB at 16 GHz and an effective absorption bandwidth (*EAB_max_*) of 5 GHz [14]. Similarly, significant progress has been made in carbon-based absorbing materials, such as reduced graphene oxide (rGO) and nano-loaded carbon materials [15]. For instance, Ning, M., and others reported a material consisting of ultrathin MoS_2_ nanosheets encapsulated in hollow carbon spheres, which significantly enhanced microwave absorption efficiency [16]. Additionally, non-metallic 2D carbon material/magnetic particle composites have proven to be efficient broadband electromagnetic wave absorbers due to their unique structure and electromagnetic parameters [17]. For example, a nanocapsule structure formed by combining g-C_3_N_4_ with metal nanoparticles (such as CoNi) demonstrated an effective absorption bandwidth (*RL* < −10 dB) of 3.5 GHz [18].

However, these wave-absorbing agents often rely on high-density matrix materials, such as metals or heavily filled polymer matrices, which not only increase the overall material density but also limit their suitability for lightweight and flexible applications [19,20,21,22,23]. Lightweight wave-absorbing materials require matrices that not only provide structural support but also significantly reduce density and enhance the dispersion and synergy of the absorbing agents [24,25,26,27].

In recent years, the trend has shifted toward lightweight mesoporous materials based on polymers [28,29,30,31,32], such as epoxy resin. These materials not only exhibit low density and excellent mechanical properties but also allow for the formation of complex porous structures through foaming techniques [33]. Unlike traditional foaming methods, the electromagnetic absorption field predominantly uses nucleation-free foaming technology, such as supercritical foaming, which can introduce uniformly distributed pores into the polymer matrix, increasing the multiple reflections and scattering of electromagnetic waves within the material [34,35]. For example, researchers have used nucleation-free foaming to prepare PVDF/MWCNT/graphene composites, which are important for ensuring the safe and efficient use of electronic devices [36]. However, the need for precise control of the process parameters and the high costs involved in the process significantly limits large-scale industrial applications [37]. Researchers are working to simplify nucleation-free foaming techniques [38,39], design functionalized wave-absorbing agents to improve compatibility and absorption efficiency, and build hierarchical porous structures to achieve multi-performance optimization.

Based on this, the present study employs nucleation-free foaming to construct porous structures. Unlike traditional nucleating agents, nucleation-free foaming directly forms pores in the epoxy resin matrix by controlling the gas generation and release process. During this process, carbon-based materials and ferrite wave-absorbing agents are introduced to further optimize the absorption performance. By fine-tuning the nucleation-free foaming process and the distribution of absorbing agents, this study achieves a lightweight design and multi-performance synergy optimization.

## 2. Materials and Methods

### 2.1. Materials

The reagents used in this study include epoxy resin E51 (epoxy groups and bisphenol A composition, Nantong Star Synthetic Materials Co., Ltd. (Nantong, China), analytical grade), epoxy resin curing agent D230 (O,O’-Bis(2-aminopropyl)polypropyleneglycol, Kunshan Jiulimei Electronic Materials Co., Ltd. (Kunshan, China), analytical grade), carbon dioxide (Shandong Chenyan Industrial Technology Co., Ltd. (Jinan, China), 99.9% purity), melamine (Shanghai Aladdin Biochemical Technology Co., Ltd.(Shanghai, China) 99.9% purity), Ni(CH_3_COO)_2_·4H_2_O (Shanghai Aladdin Biochemical Technology Co., Ltd. (Shanghai, China), 99.0% purity), ferric citrate (Shanghai Aladdin Biochemical Technology Co., Ltd. (Shanghai, China), analytical grade), ferrite wave absorber (Nanjing Aipu Hui Technology Co., Ltd., (Nanjing, China), analytical grade), and fumed silica (Shanghai Aladdin Biochemical Technology Co., Ltd. (Shanghai, China), analytical grade). Additionally, transparent epoxy potting adhesives A and B (Foshan Xinboqiao Electronics Co., Ltd. (Foshan, China)), 1,3-cyclohexanediamine (Zhengzhou Wubaotong Trading Co., Ltd. (Zhengzhou, China), analytical grade), and 1,4-dicyclohexane (Shanghai Aladdin Biochemical Technology Co., Ltd. (Shanghai, China), >98.0% purity) were used.

### 2.2. Formulation and Preparation of LFAMs

#### 2.2.1. Preparation of Carbon-Based Wave Absorber

Fe–Ni–C composite absorbers were prepared using ferric citrate and nickel acetate tetrahydrate (Ni(CH_3_COO)_2_·4H_2_O) as precursors and melamine as a carbon and nitrogen source through solution-based ultrasonic dispersion, low-temperature drying, and high-temperature calcination in a tube furnace. First, ferric citrate and nickel acetate were dissolved in deionized water at a molar ratio of 1:1 to form a homogeneous precursor solution. Melamine was then added to the solution, and the mixture was stirred to form a stable suspension, followed by ultrasonic treatment for 30 min to ensure thorough dispersion of all components. The resulting mixture was dried at 80 °C to obtain a solid precursor powder. The dried precursor was placed in a tube furnace and heated under a nitrogen atmosphere at a ramp rate of 5 °C/min to 600 °C, where it was held for 2 h. During the high-temperature process, melamine decomposed to generate nitrogen-doped carbon, which combined with metal ions to form a uniform composite structure.

#### 2.2.2. Epoxy Resin Initial Pre-Curing with CO_2_

The preparation process begins with pre-polymerization, in which a specified amount of epoxy resin E51 and curing agent D230 is measured and placed in an ultrasonic cleaner at 30 °C for thorough mixing via ultrasonic agitation. After pre-polymerization, primary curing is conducted, and carbon dioxide is introduced into the mixture through a steel pipette at a controlled gas intake rate, with close observation. When the sample no longer produces white flocculent precipitates, indicating complete reaction between the CO_2_ and curing agent, the CO_2_ introduction is halted.

#### 2.2.3. Secondary Curing Preparation of LFAMs Samples

**Figure 1:**
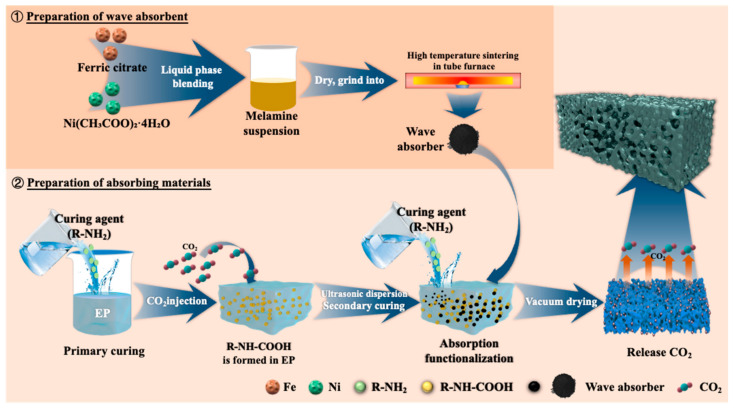
Absorbent and epoxy coreless foam preparation process.

The process continues with pre-heating treatment, in which additional epoxy resin is added to the sample according to the resin-to-curing-agent ratio (e.g., 2:1 or 3:1). The mixture is stirred to dissolve the previously formed white flocculent precipitates in the resin, after which a wave absorber is added to enhance the material’s absorption performance. This mixture is then placed in an ultrasonic cleaner at 30 °C, sealed, and ultrasonically mixed to ensure uniform dispersion of all components and the initial formation of a micro-porous structure. Following this, secondary curing is conducted by monitoring the viscosity, and once it reaches an adequate level, the sample is placed in a vacuum drying oven. Vacuum treatment is applied, allowing the sample to foam at 80 °C under vacuum, ultimately yielding an electromagnetic composite material with a microporous structure, The preparation process of the wave absorber and epoxy resin nucleation-free foaming is shown in Figure 1, and the specific formulation design is detailed in Table 1.

### 2.3. Characterization

#### 2.3.1. Scanning Electron Microscope (SEM)

The cross-sectional morphology, particle size, and distribution of wave absorbers within the epoxy resin matrix were analyzed using an SEM. It was observed that the samples, when placed on the side of the electron microscope stage and coated with gold, exhibited detailed microstructures under an acceleration voltage of 10 kV and magnification ranging from 500× to 2000×.

#### 2.3.2. Specific Surface Area Analysis (BET)

The pore structure of the epoxy foam-based wave-absorbing materials was characterized using the Kubo ×1000 specific surface area analyzer from Beijing Beode Electronics Co., Ltd. (Beijing, China). During the testing process, a certain amount of nitrogen gas was adsorbed onto the surface of the epoxy foam material.

#### 2.3.3. X-Ray Diffraction (XRD)

The thermal properties and crystal structure of the materials were assessed to identify the compounds, either crystalline or amorphous substances, using XRD with the Diffraktometer D8 from Bruker Technology Co., Ltd. (Shanghai, China). The analysis utilized a copper target to generate Cu Kα radiation (wavelength λ = 1.5406 Å), with a scanning range from 15° to 90° and a scanning speed of 10° per minute.

#### 2.3.4. Fourier Transform Infrared (FTIR)

The formation of ammonium salts during the preparation process was analyzed using the FTIR spectrometer to investigate the foaming mechanism of CO_2_ in the epoxy foam. Utilizing the FTIR spectrometer from Thermo Fisher Scientific (Waltham, MA, USA), the analysis was conducted using the attenuated total reflection (ATR) method. The experimental parameters were set as follows: a resolution of 4 cm^−1^, 30 scans, and a scanning range spanning from 400 cm^−1^ to 4000 cm^−1^.

#### 2.3.5. Vector Network Analyzer (VNA)

The real permittivity (ε′), imaginary permittivity (ε″), real permeability (μ′), and imaginary permeability (μ″) of the epoxy foam-based wave-absorbing materials were measured using the Agilent Technologies E8363B PNA vector network analyzer (VNA) (Keysight Technologies, Santa Rosa, CA, USA), over the 2–18 GHz frequency range. The samples were prepared in a coaxial ring shape, with an outer diameter of 7.0 mm, an inner diameter of 3.5 mm, and a thickness of 2.0–3.0 mm.

#### 2.3.6. Differential Scanning Calorimetry (DSC)

The thermal properties and crystal structure of the materials were analyzed to measure the structure and thermal stability of the composite materials using the DSC 200 F3 from Netzsch Instrument Manufacturing Co. (Selb, Germany). The analysis was performed under a nitrogen atmosphere with a heating rate of 10 °C/min, covering a temperature range from 30 °C to 250 °C.

## 3. Results

### 3.1. Microstructure and Morphology Analysis

**Figure 2:**
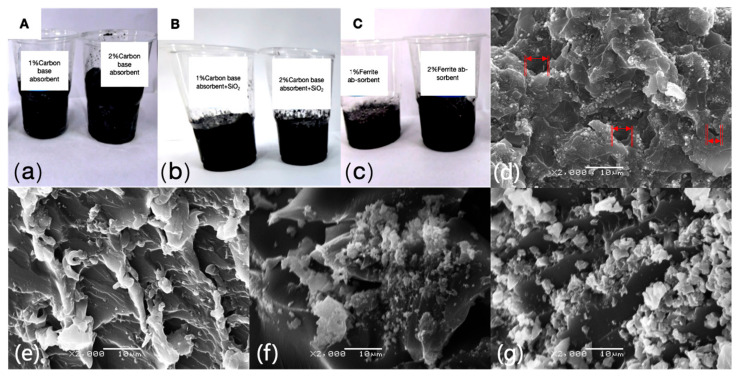
(**a**–**c**) Foaming volume comparison of LFAMs via **SEM:** (**d**,**e**) LFAMs–A2 ×2000; (**f**) LFAMs–B2 ×2000; (**g**) LFAMs–C2 ×2000.

The microscopic structure observed through SEM reveals that all samples exhibit varying degrees of porous features. Notably, Figure 2d,e shows that the A2 sample, with the addition of 2.0 wt% carbon-based absorbing agents, forms a typical porous structure with pore sizes ranging from 3 to 8 microns, consisting of voids and cracks of different sizes, and these pores are relatively uniformly distributed. Similarly, in the samples with 2.0 wt% carbon-based absorbing agents + SiO_2_ (LFAMs–B2) and 2.0 wt% ferrite absorbing agents (LFAMs–C2), similar porous structures can be observed (Figure 2f,g). These porous structures help enhance electromagnetic wave attenuation through multiple reflections and scattering, thereby improving the material’s wave absorption capability.

Further analysis of the pore structure’s boundary layer reveals irregularly shaped and sized particles, which may correspond to the deposition or local aggregation of the absorbing agents. This further confirms the successful dispersion and effective incorporation of the absorbing agents into the epoxy matrix [40]. Fine cracks or layered structures can also be observed between these particles, whose surfaces are rough and exhibit uneven textures. This significantly increases the roughness of the pore walls, further enhancing the interactions between the electromagnetic waves and the pore walls, thereby promoting the absorption of electromagnetic wave energy.

It is worth noting that these particles likely comprise uniformly dispersed SiO_2_ or ferrite. Although the addition of silica particles provides more nucleation sites, it also occupies part of the matrix volume, reducing the available space for bubble expansion, which in turn lowers the foam expansion volume [41]. Moreover, the incorporation of silica may increase the viscosity of the matrix, thus restricting the expansion and growth of bubbles (see Table 1, foam volume).

Overall, this porous structure suggests that during the nucleation-free foaming process, the absorbing agents may act as nucleating agents, significantly promoting the formation of uniform pores. This mechanism not only facilitates more effective bubble distribution during foaming but may also optimize the size and morphology of the pores, providing important support for the fine-tuning of the material’s microstructure.

### 3.2. Pore Structure and Specific Surface Area Characterization

**Figure 3:**
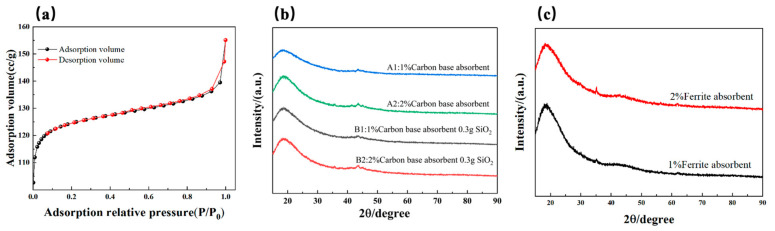
(**a**) Isothermal adsorption curve of the sample: (**b**) A1/2, B1/2 XRD pattern; (**c**) C1/2 XRD pattern.

The pore distribution verified through BET reveals the presence of a hierarchical porous structure within the LFAMs (Figure 3a). In the low relative pressure region (P/P_0_ < 0.2), the material exhibits an adsorption volume of 110–120 cc/g, indicating the uniform diffusion of CO_2_ generated during the reaction and the formation of stable micropores. In the medium relative pressure region (P/P_0_ = 0.2–0.8), the adsorption volume gradually increases to 130–140 cc/g, suggesting the presence of abundant mesopores. At high relative pressures (P/P_0_ > 0.8), the adsorption volume rises sharply to exceed 150 cc/g, revealing the existence of a certain number of macropores.

This confirms that the carbon-based absorbers, with their high specific surface area and excellent dispersibility, not only promote bubble nucleation during the nucleation-free foaming process but also provide physical support for bubble expansion [42]. This facilitates the synergistic optimization of micropores, mesopores, and macropores. The resulting hierarchical porous structure enhances the material’s specific surface area and adsorption performance, while also providing pathways for multiple reflections and the dissipation of electromagnetic waves [43].

### 3.3. Phase Structure and Crystallization Behavior Analysis

The comparative XRD analysis reveals significant differences in diffraction characteristics between samples containing only carbon-based absorbers (LFAMs-A1/2) and those containing both carbon-based absorbers and SiO_2_ (LFAMs–B1/2). The diffraction peaks of LFAMs-A1/2 (Figure 3b) around 20° exhibit broad and weak features, indicating that the material is predominantly amorphous. With an increase in the carbon-based absorber content, the diffraction intensity slightly increases, but no significant crystalline signals are observed. In contrast, LFAMs–B1/2 (Figure 3b) show a slight increase in diffraction peak intensity in the same region, but the peak width remains largely unchanged, suggesting that the addition of SiO_2_ has limited influence on the ordering of the internal crystalline structure, and the overall phase remains amorphous.

These findings confirm that carbon-based absorbers provide stable support during the nucleation-free foaming process [44]. The amorphous structure prevents localized stress concentrations that may arise from crystalline phases, enabling a more uniform pore distribution and superior dielectric loss performance. In comparison, the introduction of SiO_2_ has a relatively small effect on improving pore uniformity and wave absorption performance. The results demonstrate that incorporating carbon-based absorbers meets the requirements of the nucleation-free foaming process, simplifying the material preparation workflow while achieving efficient structural and performance optimization [45].

### 3.4. Chemical Bonding and Foaming Mechanism Study

**Figure 4:**
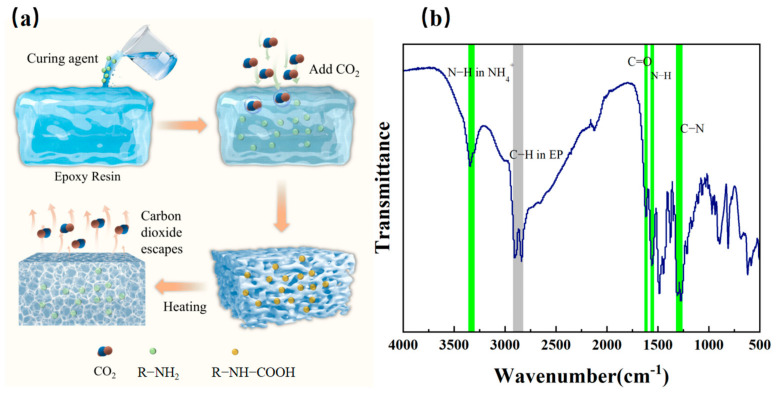
(**a**) Foaming mechanism diagram; (**b**) infrared analysis spectrum.

FTIR analysis further elucidates the gas-phase nucleation-free foaming mechanism and the reasons for the uniform distribution of pores. After the completion of primary curing, the infrared spectrum (Figure 4b) shows a broad absorption peak at 3300–3500 cm^−1^ corresponding to the stretching vibrations of the -NH groups, while the peaks at 1500–1700 cm^−1^ are attributed to the C=O bonds in the carboxylates. These characteristic absorption peaks clearly confirm the nucleophilic attack of the nitrogen atom in the amines on the electrophilic carbon atom in carbon dioxide, leading to the formation of carbamate intermediates [46]. The resulting carbamates are further stabilized through hydrogen bonding and act as microscale nucleation sites in the nucleation-free foaming system, significantly promoting the uniform generation and distribution of bubbles [47].

The study further controls gas release and bubble expansion through secondary curing. At this stage, carbon-based or ferrite absorbers are introduced. These absorbers exhibit a high specific surface area and molecular-level chemical activity, with their surfaces serving as sites for CO_2_ accumulation and initial nucleation, thereby reducing the activation energy for bubble formation. As the temperature increases to 80 °C, ammonium salts begin to decompose, releasing CO_2_, and the bubbles gradually expand to form a hierarchical porous structure. During this process, the synergistic effects of the gas release rate and changes in the matrix viscosity ensure controlled bubble growth, preventing bubble coalescence or rupture. Additionally, the absorbers are uniformly distributed within the pore walls, enhancing the mechanical stability of the material and significantly improving its broadband wave absorption performance through increased dielectric and magnetic losses. The bidirectional reaction of carbamates is as follows:

(1) $$R-NH_{2}+CO_{2}\to R-NH-COOH$$

(2) $$R-NH-COOH\overset{Heating}{\to }R-NH_{2}+CO_{2}$$

### 3.5. Electromagnetic Parameters and Wave Absorption Performance Testing

#### 3.5.1. Reflection Loss and Wave Absorption Performance

**Figure 5:**
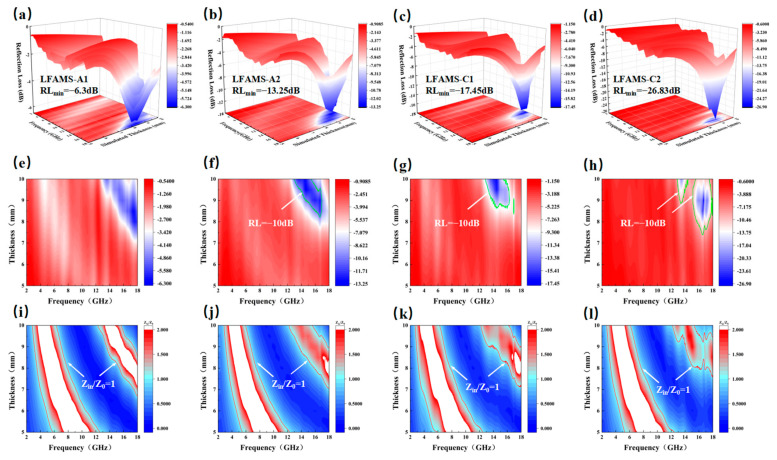
(**a**–**d**) The 3D projection of the reflection loss for LFAMs; (**e**–**h**) 2D mapping of the reflection loss for LFAMs; (**i**–**l**) 2D impedance matching of LFAMs.

To verify the functional integration of mesoporous structures with wave absorption performance, this study measured the electromagnetic parameters of LFAMs, including complex permittivity (*ε_r_ = ε′*–*jε″*) and complex permeability (*μ_r_ = μ′*–*jμ″*), using a vector network analyzer. Additionally, the dielectric loss tangent (tan *δ_ε_*), magnetic loss tangent (tan *δ_μ_*), and reflection loss (*RL*) were calculated. The calculation formulas are as follows [48]:

(3) $$\tan\delta_{\varepsilon }=\frac{\varepsilon^{\prime \prime }}{\varepsilon^{\prime }}$$

(4) $$\tan\delta_{\mu }=\frac{\mu \prime \prime }{\mu \prime }$$

(5) $$Z_{in}=Z_{0}\sqrt{\frac{\mu_{r}}{\varepsilon_{r}}}\tan h\left(j\frac{2\pi fd}{c}\right)$$

(6) $$RL=20\log\left|\frac{Z_{in}-Z_{0}}{Z_{in}-Z_{0}}\right|$$

where *Z_in_* is the input impedance, *Z_0_* is the impedance of free space, *f* is the frequency of EMW, *d* is the thickness of the material, and *c* is the speed of light. The analysis of reflection loss reflects the regulatory effect of the material’s microstructure on its performance. For the sample with 1.0 wt% carbon-based absorbers (LFAMs–A1), the *RL_min_* reaches −6.3 dB at around 17 GHz (Figure 5a), mainly driven by interfacial polarization effects and limited conductive loss mechanisms [49]. Due to the low carbon content, the conductive network is not fully formed, resulting in restricted dielectric loss capacity and weaker absorption performance in the high-frequency range. However, the uniform porous structure constructed by the nucleation-free foaming process provides a solid foundation for bubble distribution and pore wall uniformity, allowing the absorbers to disperse homogeneously. This ensures effective interfacial polarization, even with a limited absorber content. As the carbon content increases to 2.0 wt% (LFAMs–A2), the *RL_min_* decreases to −13.25 dB, and the EAB expands to 12.9–17 GHz (Figure 5b). The higher carbon content further enhances the formation of the conductive network, strengthens dielectric loss capacity, and optimizes impedance matching, ensuring efficient absorption of electromagnetic wave energy in the high-frequency range.

For the ferrite-based composite absorbers (LFAMs–C1/2), the magnetic loss mechanism is particularly prominent in the mid-to-low frequency range. For the sample with 1.0 wt% ferrite (LFAMs–C1), the *RL_min_* reaches −17.45 dB at around 14 GHz, and the EAB extends to 12.8–17 GHz (Figure 5c). This is mainly driven by the synergistic effects of magnetic hysteresis loss and eddy current loss. At this stage, the high specific surface area of the absorbers, combined with the porous structure, reduce the activation energy for bubble formation, further enhancing eddy current loss and interfacial polarization effects. When the ferrite content increases to 2.0 wt% (LFAMs–C2), the *RL_min_* further decreases to −26.83 dB at around 16.6 GHz, while the EAB extends significantly to 12.7–18 GHz (Figure 5d). This significant improvement is attributed to the magnetic hysteresis loss provided by the ferrite absorbers, as well as the synergistic effect of the mesoporous structure orientation mechanism [50]. Meanwhile, the gas-controlled release mechanism during the nucleation-free foaming process ensures uniformity in the porous structure, preventing bubble coalescence or rupture, further optimizing the matching of complex magnetic permeability and complex permittivity.

#### 3.5.2. Electromagnetic Parameters Analysis

**Figure 6:**
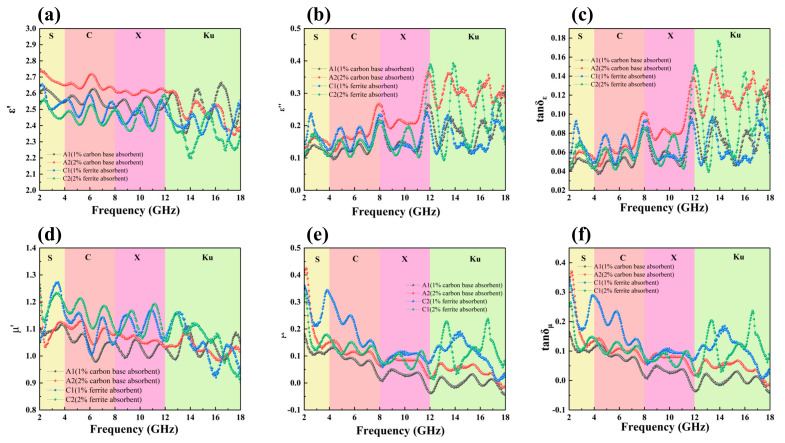
(**a**) Real complex permittivity, (**b**) imaginary complex permittivity, (**c**) dielectric loss tangents, (**d**) real complex permeability, (**e**) imaginary complex permeability, and (**f**) magnetic loss tangents.

To clarify the loss mechanisms of LFAMs, we further analyzed the dielectric constant and magnetic permeability. The real part of the dielectric constant (*ε′*) reflects the material’s ability to store electrical energy, while the imaginary part (*ε″*) is closely related to energy loss (i.e., absorption capacity). The dielectric loss tangent (*tanδ_ε_*), which is the ratio of the imaginary to the real part of the dielectric constant, characterizes the material’s energy loss properties [51]. Our results show that with the addition of carbon-based absorbers, the *ε′* of LFAMs-A2 (Figure 6a) increases significantly in the 2–14 GHz range compared to that of the LFAMs–A1, while the *ε″* (Figure 6b) of LFAMs-A2 is higher than that of LFAMs–A1 across the 2–18 GHz range. Correspondingly, *tanδ_ε_* follows the same trend as *ε″* (Figure 6c), which explains the superior *RL_min_* of LFAMs-A2 compared to that of LFAMs–A1. This indicates that the increased carbon content significantly optimizes the conductive network and microporous structure, thus enhancing the high-frequency absorption capability.

In contrast, ferrite-based LFAMs–C1/2 have lower *ε′* than LFAMs–A1/2 (Figure 6a), but LFAMs–C2 shows higher *ε″* than LFAMs–A1/2 (Figure 6b). This leads to a higher *tanδ_ε_* for LFAMs–C2 (Figure 6c). The increase in ferrite content optimizes the structure of EP, and the mesoporous structure of EP provides sufficient dielectric loss for ferrite-based absorbers dominated by magnetic loss. It is noteworthy that LFAMs–C1 has a higher *ε″* value than LFAMs–C2 in the 13–14 GHz range, but LFAMs–C2 outperforms LFAMs–C1 in other frequency ranges. This explains why the effective absorption band of LFAMs–C1 shifts to lower frequencies, while LFAMs–C2 has better overall performance.

Interestingly, we found that the *tanδ_ε_* of LFAMs–A2 is significantly higher than that of LFAMs–C1 and, in some frequency ranges, higher than LFAMs–C2. However, when considering *RL_min_* and *EAB_max_*, LFAMs–C1/2 outperform LFAMs–A2. This can be explained by the complex permeability analysis. The real part of magnetic permeability (*μ′*) represents the material’s ability to store magnetic energy, while the imaginary part (*μ″*) is related to magnetic energy loss (i.e., magnetic wave absorption capacity). The magnetic loss tangent (*tanδ_μ_*) reflects the material’s magnetic energy loss ability under the influence of a magnetic field, and a higher value indicates stronger magnetic wave absorption capacity [52]. LFAMs–C1/2 have higher *μ′* and *μ″* values than do LFAMs–A1/2 in the 2–18 GHz range, which results in a higher *tanδ_μ_* for LFAMs–C1/2 (Figure 6d–f). This is primarily attributed to the magnetic loss capacity provided by ferrite.

#### 3.5.3. Loss Mechanisms and Performance Comparison

**Figure 7:**
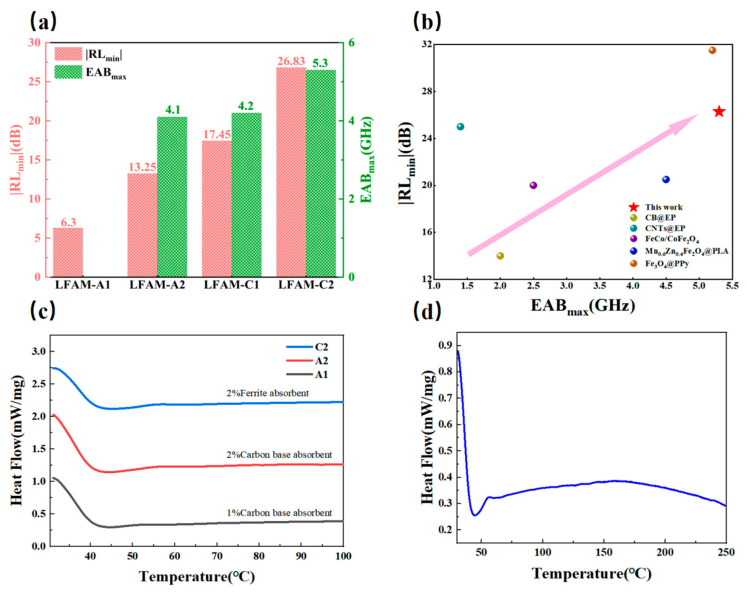
(**a**) Comparison of *RL_min_* and *EAB_ma_*_x_ for LFAMs; (**b**) Comparison of LFAMs with Existing Materials Performance; (**c**) Thermal stability curve of LFAMs–A1/2/C2 (30–100 °C); (**d**) thermal stability curve of LFAMs–C2 (30–250 °C).

We observed that the wave absorption performance of the four samples follows the same trend as *tanδ_μ_* (Figure 7a), indicating that the loss mechanism of LFAMs is primarily driven by magnetic loss, with dielectric loss from the mesoporous structure as a supplementary mechanism [53]. From the perspective of impedance matching (*Z_in_/Z*_0_), *Z_in_/Z*_0_ reflects the energy transfer efficiency between the material and the external electromagnetic wave, minimizing reflection and maximizing absorption [54]. The mesoporous structure of EP further optimizes the *Z_in_/Z*_0_ of LFAMs. In Figure 5i–l, we marked *Z_in_/Z*_0_ = 1 with a red line to analyze the ideal impedance region (0.5 ≤ *|Z_in_/Z*_0_*|* ≤ 1.5) [55]. It is clearly shown that LFAMs–A2 outperforms LFAMs–A1, and LFAMs–C2 outperforms LFAMs–C1, which further validates the optimization effect of the absorber on the mesoporous structure of EP and highlights the role of structural orientation losses in optimizing *Z_in_/Z*_0_. This demonstrates that our work successfully couples three mechanisms—dielectric loss, magnetic loss, and structural orientation loss—leading to a comprehensive improvement in performance.

By comparing this work with similar materials employed in the recent literature (Figure 7b), we found that, compared to wave-absorbing materials compounded with EP, CB@EP exhibits the *RL_min_* of −14 dB and the *EAB_max_* of 2 GHz [56], while CNTs@EP has the *RL_min_* of up to −25 dB, but its *EAB_max_* is only 1.4 GHz [57]. Compared with other ferrite-based wave-absorbing materials, FeCo/CoFe_2_O_4_ shows an *RL_min_* of −20 dB and an *EAB_max_* of 2.5 GHz [58]. In other studies, polymer composites like Mn_0.6_Zn_0.4_Fe_2_O_4_@PLA have an *RL_min_* of −20.5 dB and an *EAB_max_* of 4.5 GHz [59]. In comparison, this work shows significant improvements in both aspects. Notably, Fe_3_O_4_@PPy has an *RL_min_* of −31.5 dB, but its *EAB_max_* remains lower than that of this study, mainly due to the high conductivity of PPy [60]. This work will further enhance the electrical properties of the system to achieve further performance improvements.

### 3.6. Thermal Stability Analysis

The carbon-based absorber composite epoxy foam material exhibits stable thermal effects in the temperature range of 30 °C to 150 °C. The thermal flow of the 1.0 wt% and 2.0 wt% carbon-based absorbers (Figure 7c) gradually increases with rising temperature and maintains a high level in the mid-to-high temperature range (100 °C to 150 °C). This thermal effect is attributed to the high specific surface area and excellent thermal conductivity provided by the foamed epoxy resin, which effectively promotes internal heat conduction and dispersion within the composite material [61]. Moreover, increasing the carbon-based absorber content further enhances the material’s thermal stability and thermal effects, although the enhancement may not be linear. This indicates that carbon-based absorbers possess significant thermal stability and wave absorption performance under high-temperature conditions, making them suitable for applications in wave-absorbing materials requiring stable thermal effects at elevated temperatures.

The ferrite absorber composite epoxy foam material exhibits stable thermal effects in the low-to-medium temperature range (30 °C to 150 °C). The thermal flow initially decreases but gradually rises (Figure 7c), forming a plateau between 100 °C and 150 °C. Within this temperature range, the material demonstrates high thermal stability, with ferrite absorbers effectively stabilizing thermal flow through interfacial interactions with the epoxy matrix. The stability of this plateau region is likely associated with the magnetic loss mechanism, which is the primary wave absorption mechanism of ferrite absorbers.

In the high-temperature range (150 °C to 250 °C), the thermal flow shows a slow downward trend (Figure 7d). The thermal flow curve indicates that the material retains a certain degree of thermal stability within this temperature range. Based on the continuity of the thermal flow trend, it can be inferred that the internal thermal flow remains relatively uniform. This sustained change in thermal flow suggests that ferrite absorbers continue to participate in the material’s heat absorption and dispersion process at high temperatures. Due to the excellent magnetic loss properties of ferrite materials, they can continuously absorb electromagnetic energy through mechanisms such as hysteresis loss and eddy current loss under high-temperature environments.

## 4. Conclusions

In this study, lightweight epoxy foam electromagnetic wave-absorbing materials were successfully fabricated using a nucleation-free foaming process, overcoming the uneven foaming issues caused by traditional nucleating agents. The multi-loss mechanisms of epoxy-based wave-absorbing materials were optimized, addressing the high density and narrow absorption bandwidth issues of conventional wave-absorbing materials. This paper systematically analyzed the chemical mechanisms, microstructure, thermal stability, and wave absorption performance of the materials. Fourier-transform infrared spectroscopy (FTIR) confirmed that a nucleophilic addition reaction occurred between the curing agent, CO_2_, and water to form ammonium salts. This reaction can be reversed by thermal decomposition, providing chemical evidence for the gas-phase nucleation mechanism. X-ray diffraction (XRD) analysis showed that the materials predominantly existed in an amorphous state, lacking long-range ordered crystalline structures. The addition of carbon-based wave-absorbing agents slightly affected the local structure but maintained the amorphous characteristics, thus avoiding stress concentration from the crystalline phases and ensuring uniform pore distribution.

The epoxy foam materials exhibited excellent thermal stability, with minimal influence from the type and content of the wave-absorbing agents, indicating their suitability for high-temperature environments. Scanning electron microscopy (SEM) analysis confirmed the uniform embedding of wave-absorbing agents within the epoxy matrix, optimizing pore distribution and further validating the feasibility of the nucleation-free foaming process. Notably, the addition of 2.0 wt% carbon-based wave-absorbing agents led to the formation of a uniform hierarchical pore structure, expanding the material’s volume to 4.6 times its original size, achieving a lightweight design. Epoxy foam materials with carbon-based wave-absorbing agents (LFAMs–A2) achieved a minimum reflection loss (RL) of −13.25 dB and an effective absorption bandwidth (EAB) of 13–17 GHz. Epoxy foam materials with ferrite-based wave-absorbing agents (LFAMs–C2) demonstrated exceptional electromagnetic wave absorption performance at 16.6 GHz, with a minimum reflection loss (RL) of −26.83 dB and an effective absorption bandwidth (EAB) of 5.3 GHz, nearly covering the Ku band. 

Furthermore, this study provides a new approach for lightweight coatings and structural optimization through the nucleation-free foaming process, enhancing both structural orientation loss and distribution optimization. The proposed novel matrix preparation process supports research on structural–functional integration and is expected to significantly promote the lightweighting of thick coatings, particularly in fields such as aerospace, military protection, communication devices, and automotive electronics, thereby greatly enhancing the functionality and performance of these devices.

## Figures and Tables

**Table 1:** Experimental formulation design and foam volume of LFAMs.

Sample	CO_2_/L	SiO_2_/g	Carbon Base Absorbent Content/%	Ferrite Absorbent Content/%	Foaming Volume/Times
A1	0.24	0	1.0	0	4.0
A2	0.24	0	2.0	0	4.6
B1	0.24	0.3	1.0	0	3.3
B2	0.24	0.3	2.0	0	3.0
C1	0.24	0	0	1.0	2.2
C2	0.24	0	0	2.0	3.5

## Data Availability

The data presented in this study are available on request from the corresponding author.
